# Different regulatory mechanisms of hepatitis B- and C-positive hepatocellular carcinoma by single-cell RNA sequencing

**DOI:** 10.1016/j.gendis.2026.102289

**Published:** 2026-06-09

**Authors:** Su-Jin Baek, Seung-Jin Park, Minho Lee, Won Park, Jong-Hwan Kim, Youngjin Shin, Ok-Hee Kim, Ho Joong Choi, Say-June Kim, Yeun-Jun Chung, Seon-Young Kim

**Affiliations:** a KM Data Division, Korea Institute of Oriental Medicine, 1672 Yuseong-daero, Yuseong-gu, Daejeon 34054, Republic of Korea; b Korea Research Institute of Bioscience and Biotechnology (KRIBB), Daejeon 34131, Republic of Korea; c Department of Bioscience, University of Science and Technology (UST), Daejeon 34113, Republic of Korea; d Department of Life Science, Dongguk University-Seoul, 32 Dongguk-ro, Ilsandong-gu, Goyang-si, Gyeonggi-do 10326, Republic of Korea; e Themoagen Inc, Daejeon 35868, Republic of Korea; f Korea Bioinformation Center (KOBIC), Korea Research Institute of Bioscience and Biotechnology (KRIBB), Daejeon 34131, Republic of Korea; g Department of Surgery, Seoul St. Mary's Hospital, College of Medicine, The Catholic University of Korea, Seoul 06591, Republic of Korea; h Catholic Central Laboratory of Surgery, Institute of Biomedical Industry, College of Medicine, The Catholic University of Korea, Seoul 06591, Republic of Korea; i Department of Biomedicine & Health Sciences, College of Medicine, The Catholic University of Korea, Seoul 06591, Republic of Korea; j Departments of Microbiology, IRCGP, College of Medicine, The Catholic University of Korea, Seoul 06591, Republic of Korea; k Precision Medicine Research Center, College of Medicine, The Catholic University of Korea, Seoul 06591, Republic of Korea

Hepatocellular carcinoma (HCC) is the third leading cause of cancer-related deaths worldwide. It accounts for nearly 90% of all primary liver cancers. The incidence of HCC is primarily driven by chronic hepatitis B virus (HBV) and hepatitis C virus (HCV) infections, which together are responsible for approximately 80% of global cases.^1^^,^^2^ HBV, a DNA virus, and HCV, an RNA virus, possess fundamentally different genomic structures and replication strategies. This suggests they may trigger carcinogenesis through distinct molecular pathways. However, virus-specific molecular distinctions in HCC pathogenesis remain poorly defined. Historically, bulk RNA sequencing has obscured critical cellular heterogeneity by averaging signals across mixed cell populations. The advent of single-cell RNA sequencing (scRNA-seq) has overcome this limitation, enabling high-resolution profiling of individual cells. While scRNA-seq has been applied to HCC, for instance, to profile the diverse immune cell subsets within the HBV/HCV microenvironment,^3^ and to identify unique, immunosuppressive tumor cell phenotypes specific to HBV-HCC,^4^ a systematic comparison of their distinct cellular ecosystems remains poorly characterized. To fill this gap, we conducted an integrative scRNA-seq analysis to systematically investigate the distinct molecular characteristics of HBV- and HCV-HCC. We integrated data from normal, HBV- and HCV-HCC tissues to identify cell-type-specific transcriptional alterations.

We constructed a comprehensive single-cell atlas by integrating data from 51 samples, comprising 23 HBV-HCC patients, 19 HCV-HCC patients, and 9 normal liver controls (Fig. 1A and Table S1). Patients with known HBV/HCV co-infection were excluded from this cohort. A total of 196,658 high-quality cells were retained for analysis. These cells were clustered using the top 20 principal components and visualized via UMAP (Fig. 1B). Using established canonical markers, we identified 48 cell clusters annotated into 10 major cell types (Fig. 1C; Fig. S1 and Table S2). Additionally, due to the distinct distributions of the Normal, HBV, and HCV groups, the proportions of cell types also varied significantly across the datasets (Fig. S2). Comparison of cell types within each group revealed that the most abundant populations across all samples were T cells (68,684 cells; 34.93%) and hepatocytes (73,745 cells; 37.5%) (Fig. 1D). Normal liver samples had a higher proportion of hepatocytes, whereas both HBV- and HCV-HCCs showed a marked decrease in hepatocytes and a corresponding increase in T cells. Critically, we observed a virus-specific difference: the proportion of cholangiocytes was higher in HCV-HCC (2%) compared with HBV-HCC (1%) (Fig. 1E). Furthermore, differentially expressed gene analysis comparing the viral subtypes revealed that cholangiocytes, along with smooth muscle cells and neutrophils, showed a high number of differentially expressed genes, underscoring their significant transcriptional divergence (Fig. S3 and Table S3). Given their high transcriptional divergence, we focused on cholangiocytes to uncover virus-specific mechanisms.

**Figure 1:**
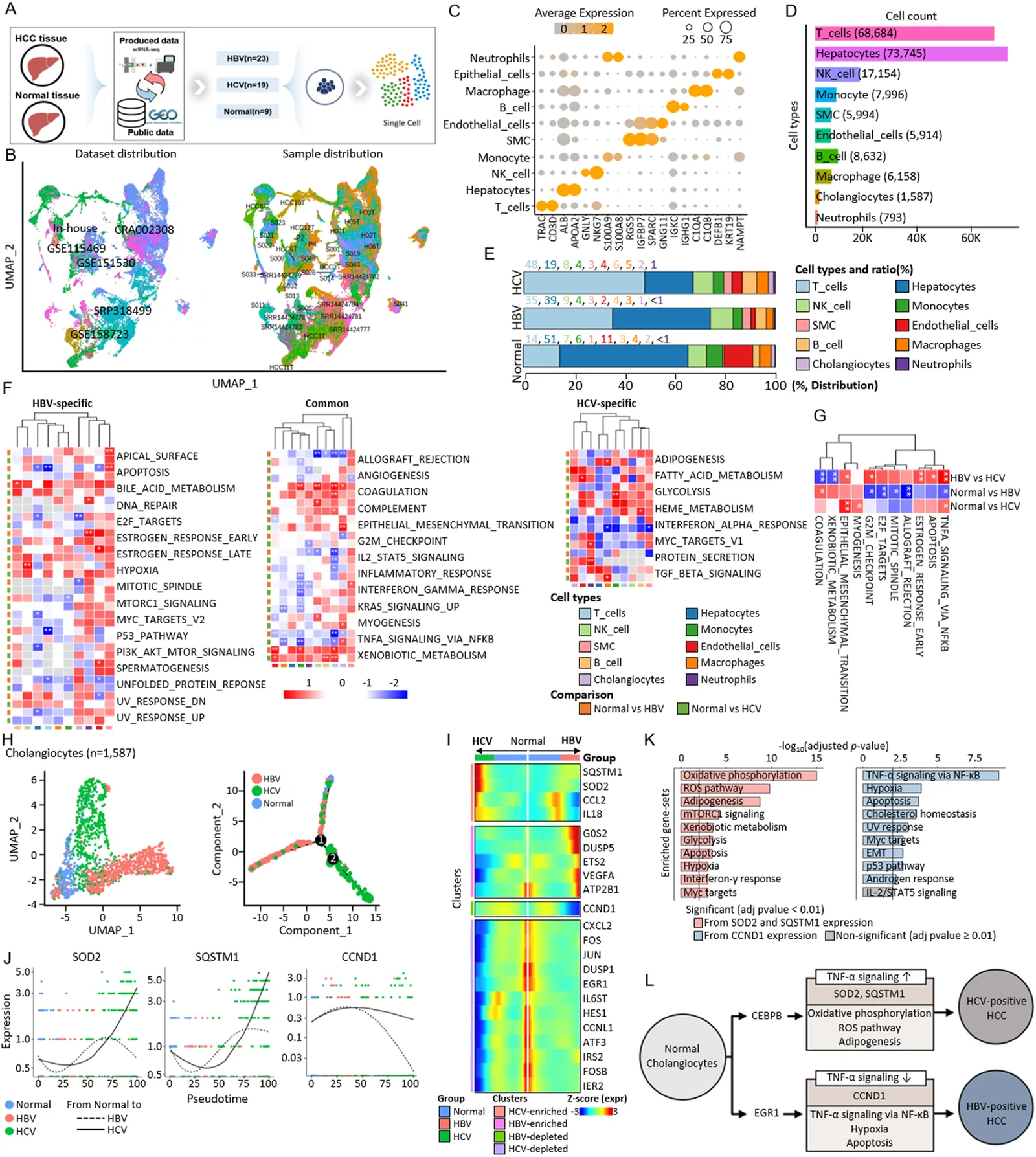
Virus-specific transcriptional landscapes of HBV- and HCV-positive hepatocellular carcinoma revealed by single-cell RNA sequencing. **(A)** Schematic representation of the workflow; 9 normal and 23 HBV- and 19 HCV-HCC patient datasets were obtained from 51 samples. **(B)** UMAP plot of 196,658 cells from 51 samples. The left panel is colored by six datasets, and the right panel is colored by individual samples. **(C)** Average gene expression of selected marker genes for each major cell type. One or two cell-type marker genes were selected with the most significant expression for each of the ten major cell types. **(D)** Cell count for each major cell type. **(E)** Distribution of major cell types for each group (Normal, HBV, and HCV). The numbers above the bars indicate the specific percentage of each cell type. **(F)** Comparison of major pathways between HBV- and HCV-HCC. Classification of major pathways by each condition. Heatmap of HBV-specific pathway group. HBV-specific group represents significant pathways only in HBV-positive samples (left panel). Heatmap of common pathway group between HBV-positive and HCV-positive samples (middle panel). Heatmap of HCV-specific group (right panel). HBV-specific, HCV-specific, and common groups indicate pathways significant in only HBV-HCC, only HCV-HCC, or both, respectively. ∗*p* < 0.05 and ∗∗*p* < 0.01. **(G)** Heatmap of enriched pathways in cholangiocytes. **(H)** UMAP clustering (left) and trajectory plot based on pseudotime analysis (right) in cholangiocytes. **(I)** A heatmap showing 22 branch-dependent regulated genes from the BEAM function belonging to TNF-a signaling. Genes were classified into four clusters showing increased (HBV-enriched/HCV-enriched) or decreased (HBV-depleted/HCV-depleted) expression along pseudotime from normal to HBV or HCV. We set a cut-off FDR value of < 0.05. **(J)** Line plots showing the expression changes of three key genes (SOD2, SQSTM1, CCND1) according to pseudotime. Each line indicates the group: Normal (gray), HBV (red), and HCV (blue). **(K)** Results of gene-set enrichment analysis. (Left) Based on 168 genes highly correlated with SOD2 and SQSTM1. (Right) Based on 100 genes with a high correlation with CCND1. **(L)** Summary diagram of this study.

To understand the functional consequences of these transcriptional changes, we performed Gene Set Enrichment Analysis (GSEA) across all cell types using the comprehensive Hallmark gene set. This analysis allowed us to categorize key dysregulated pathways into three groups: HBV-specific, HCV-specific, and common, representing those significant in HBV-HCC only, HCV-HCC only, or in both types. HBV-specific pathways included down-regulated "E2F TARGETS" and "P53 PATHWAY", and up-regulated "BILE ACID METABOLISM" and "HYPOXIA". HCV-specific pathways included up-regulated "ADIPOGENESIS" and "TGF BETA SIGNALING", and down-regulated "INTERFERON ALPHA RESPONSE" (macrophages/smooth muscle cells). Common pathways included down-regulated "ANGIOGENESIS" and "IL2 STAT5 SIGNALING", and up-regulated "COAGULATION" and "XENOBIOTIC METABOLISM" (Fig. 1F; Fig. S4 and Table S4). While these broad changes are informative, a cell-type-specific GSEA highlighted our central finding. Within cholangiocytes, while most pathways showed similar enrichment patterns, the "TNFA SIGNALING VIA NFKB" pathway exhibited an opposite expression pattern (Fig. 1G). This critical inflammatory pathway was significantly decreased in HBV-positive cells but significantly increased in HCV-positive cells compared with normal cholangiocytes. We investigated the 52 differentially expressed genes within the "TNFA SIGNALING VIA NFKB" pathway and found that most genes followed this divergent pattern (Figs. S5 and S6). Key genes, including *MARCKS*, *JUN*, *EGR1*, *CCND1*, *FOS*, and *NFKBIA*, showed decreased expression in HBV-cells but increased expression in HCV-positive cells. This demonstrated a fundamental, virus-specific re-wiring of TNF-α signaling specifically within the cholangiocyte population. To assess the clinical relevance of these findings, we performed survival analysis using TCGA LIHC data, stratifying by viral status (confirmed via clinical data and read alignment) (Fig. S7). This analysis identified prognostic genes that were effective only in a virus-dependent manner. Low expression of *IFIT2*, a gene decreased in our HBV-positive data, correlated with significantly shorter overall survival only in HBV-HCC patients (*p* = 0.027). *IFIT2* limits HBV replication.^5^ Conversely, high expression of the chemokines *CXCL1* and *CXCL3*, genes up-regulated in our HCV-positive data, correlated with reduced survival only in HCV-HCC patients (*p* = 0.048 and *p* = 0.015, respectively). These findings confirm that the distinct transcriptional programs we identified have tangible, virus-specific prognostic implications.

To investigate which genes regulated this divergence, we selected cell types that best reflected this divergence, and it was observed that cholangiocytes showed the clearest relationship among the normal, HBV, and HCV groups (Fig. S8). We performed trajectory analysis on the 1587 cholangiocytes (Fig. 1H) and, when ordering cells in pseudotime, normal cholangiocytes differentiated along a trajectory that bifurcated into two distinct malignant states: an HBV-positive branch and an HCV-positive branch. We identified 22 branch-dependent genes belonging to the "TNFA SIGNALING VIA NFKB" by applying the Branched Expression Analysis Modeling (BEAM) function to detect genes with significant expression changes between the HBV-HCC and HCV-HCC branches (Fig. 1I). According to the expression pattern by pseudotime, the genes were classified into four clusters with increased (HBV-enriched)/decreased (HBV-depleted) expression when progressing from normal to HBV and increased (HCV-enriched)/decreased (HCV-depleted) expression when progressing from normal to HCV. We focused on the key clusters defining the divergence: "HBV-depleted" (*e*.*g*., *CCND1*) and "HCV-enriched" (*e*.*g*., *SOD2*, *SQSTM1*) (Fig. 1J and Table S5). We discovered CEBPB as the candidate transcription factor regulating the genes that increase from Normal to HCV (Fig. S9A). We also identified EGR1 as the candidate transcription factor regulating genes decreasing from Normal to HBV (Fig. S9B). GSEA performed on the top 100 genes highly correlated with these transcription factors (Tables S6 and S7) supported their distinct functional programs (Fig. 1K and Table S7). The EGR1–HBV axis was associated with "Hypoxia", "TNF-α signaling via NF-kB", and "Apoptosis", while the CEBPB–HCV axis was linked to "Oxidative phosphorylation", "ROS pathway", and "Adipogenesis".

In conclusion, this study provides one of the first direct scRNA-seq comparisons of HBV- and HCV-HCC, revealing distinct cellular and molecular landscapes. Our key finding is the divergent regulation of the TNF-α signaling pathway in cholangiocytes, which is down-regulated in HBV-HCC and up-regulated in HCV-HCC. We suggest this divergence may be orchestrated by distinct candidate transcription factors—EGR1 in HBV-HCC and CEBPB in HCV-HCC. These transcription factors, in turn, are associated with different target genes (*CCND1 vs*. *SOD2*/*SQSTM1)* and downstream functional pathways (hypoxia/apoptosis *vs*. oxidative phosphorylation/ROS pathway) (Fig. 1L). This suggests HBV suppresses this pathway (potentially evading apoptosis), while HCV activates it (potentially promoting chronic inflammation). These findings provide correlative insights into the divergent oncogenic roles of HBV and HCV and offer valuable, virus-specific gene signatures.

## CRediT authorship contribution statement

**Su-Jin Baek:** Writing – review & editing, Writing – original draft, Visualization, Investigation, Formal analysis, Data curation, Conceptualization. **Seung-Jin Park:** Writing – original draft, Visualization, Formal analysis, Data curation. **Minho Lee:** Writing – review & editing, Data curation, Conceptualization. **Won Park:** Writing – original draft, Visualization, Formal analysis, Data curation. **Jong-Hwan Kim:** Formal analysis, Data curation. **Youngjin Shin:** Formal analysis, Data curation. **Ok-Hee Kim:** Formal analysis. **Ho Joong Choi:** Data curation. **Say-June Kim:** Writing – review & editing, Conceptualization. **Yeun-Jun Chung:** Writing – review & editing, Conceptualization. **Seon-Young Kim:** Funding acquisition, Conceptualization.

## Funding

This work was supported by the National Research Foundation (Republic of Korea) (No. RS-2026-25486775 to S.Y.K.) through the Ministry of Science and ICT (MSIT) and the Systemic Industrial Infrastructure Projects (No. P0009796, 2019 to S.Y.K.) through the Ministry of Trade, Industry and Energy (Republic of Korea).

## Conflict of interests

The authors declared no conflict of interests.
