# Supplementary material for: Different regulatory mechanisms of hepatitis B- and C-positive hepatocellular carcinoma by single-cell RNA sequencing
Source: Genes Dis. 2026 Jun 9;14(2):102289. doi: 10.1016/j.gendis.2026.102289 (PMC13634556; doi:10.1016/j.gendis.2026.102289)
Supplement: Supplementary file 1 [file mmc3.pdf]

Supplementary Figure 1

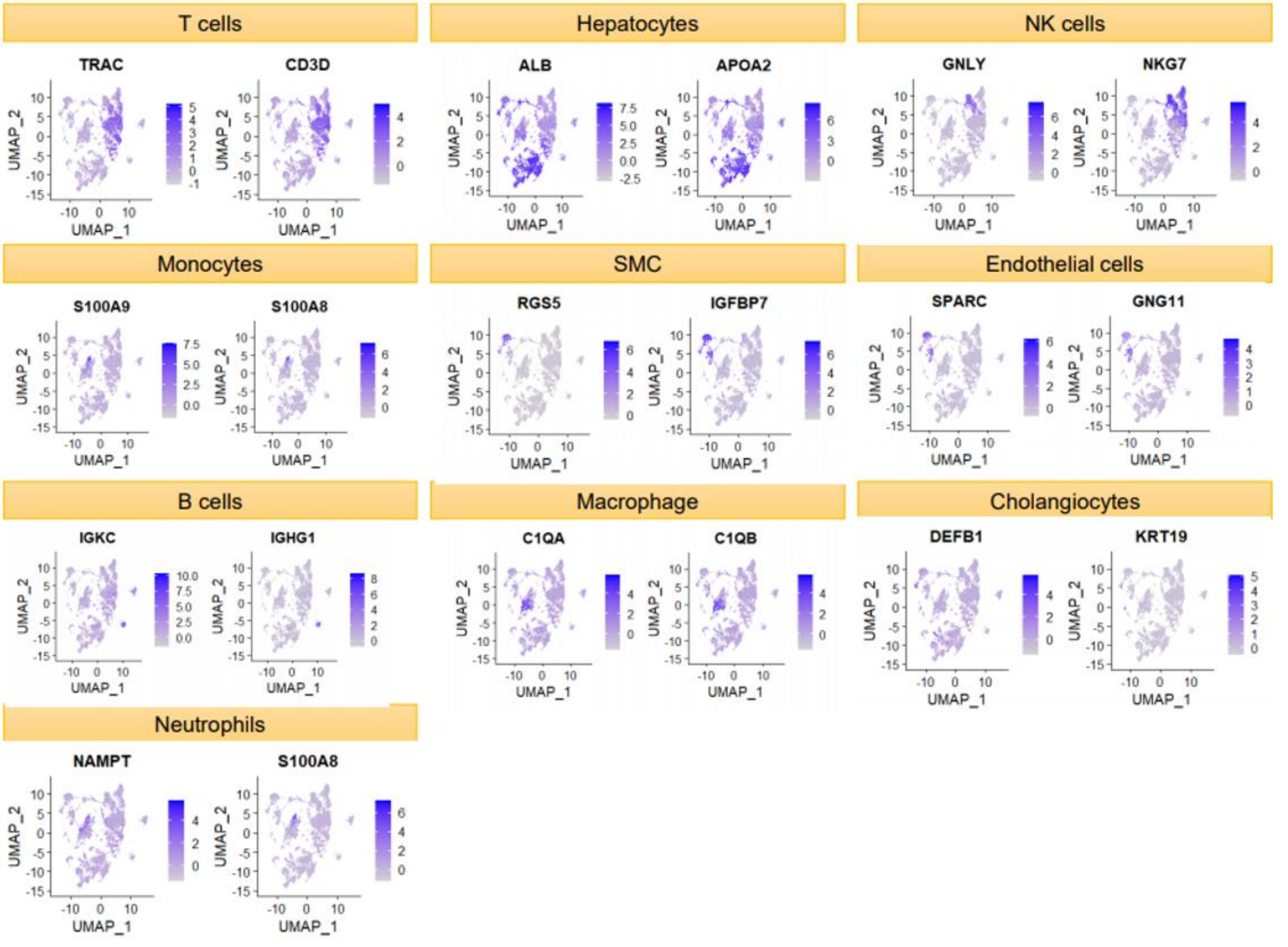

Identification of major cell type markers. Expression of marker genes across cell types.

Supplementary Figure 2

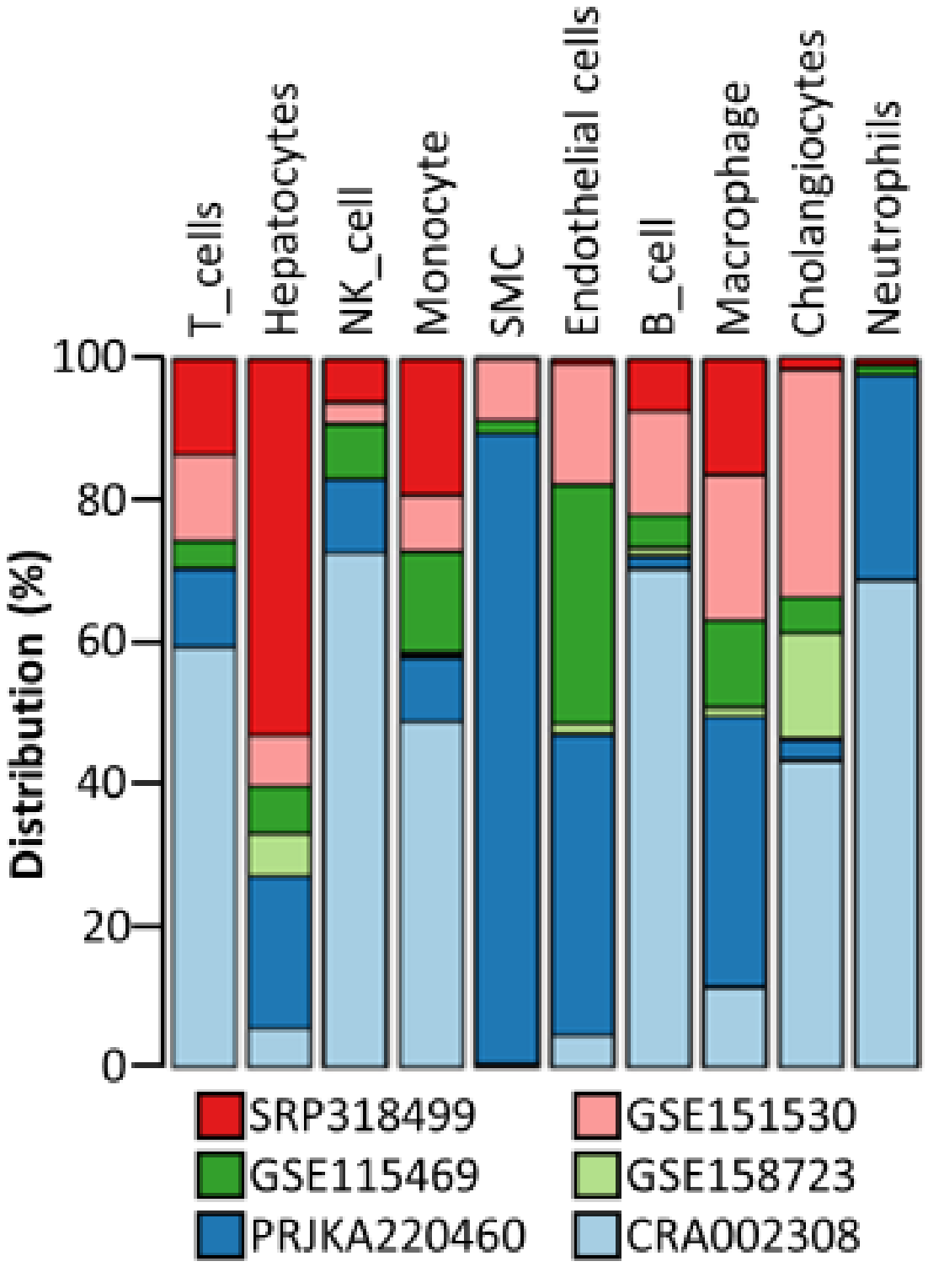

Distribution of datasets for each major cell type.

Supplementary Figure 3

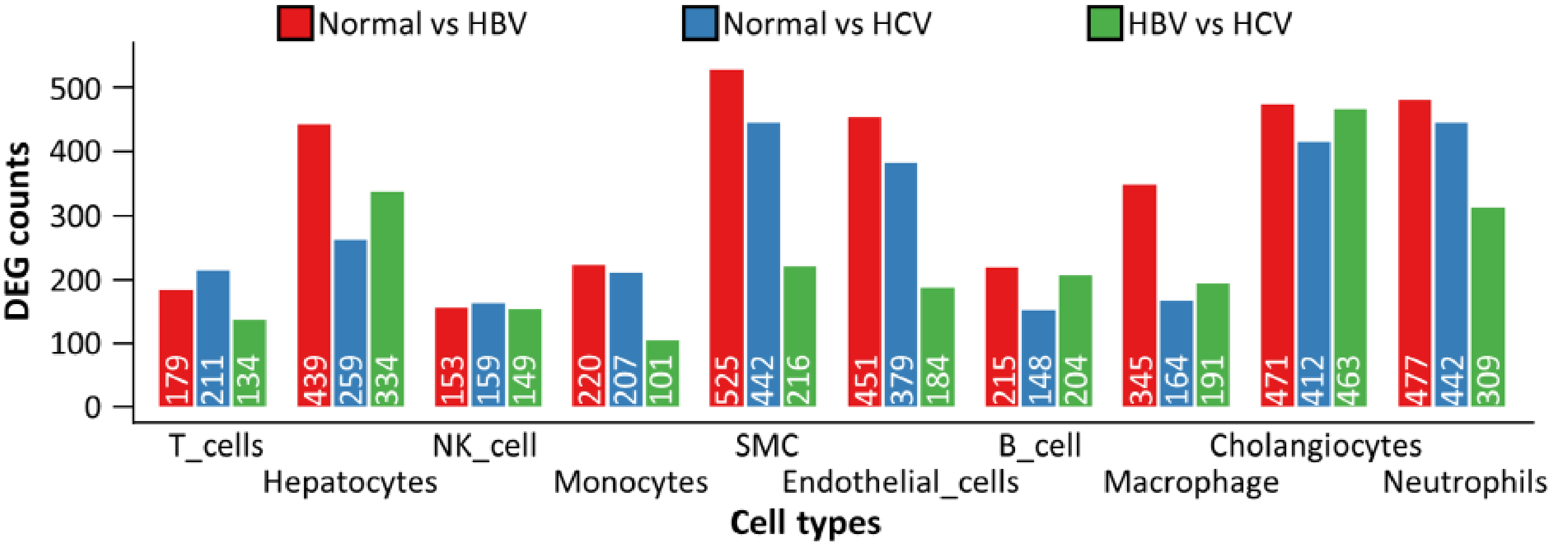

Comparison of DEG count for each condition.

Supplementary Figure 4

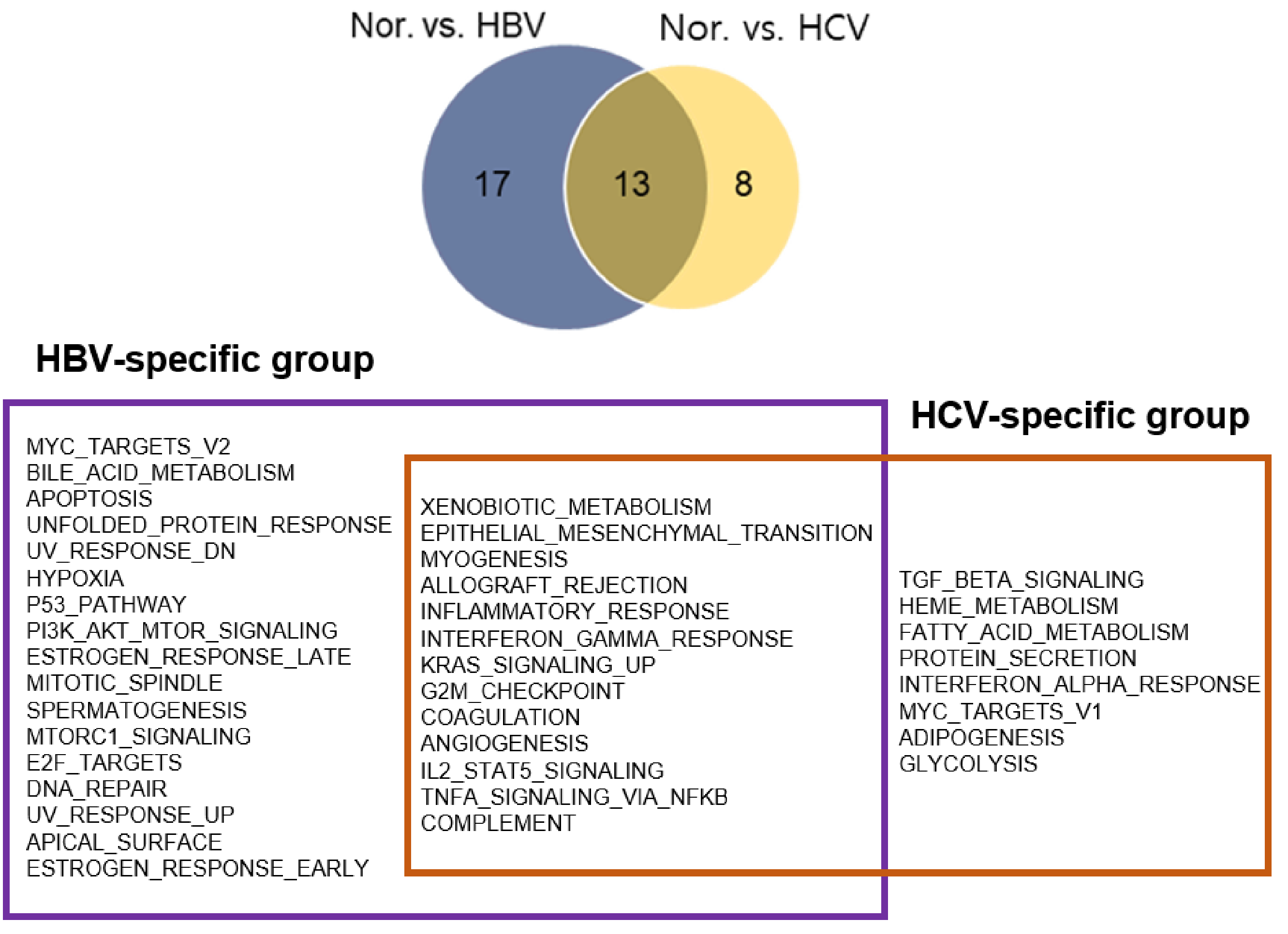

Classification of major pathways by each condition

Supplementary Figure 5

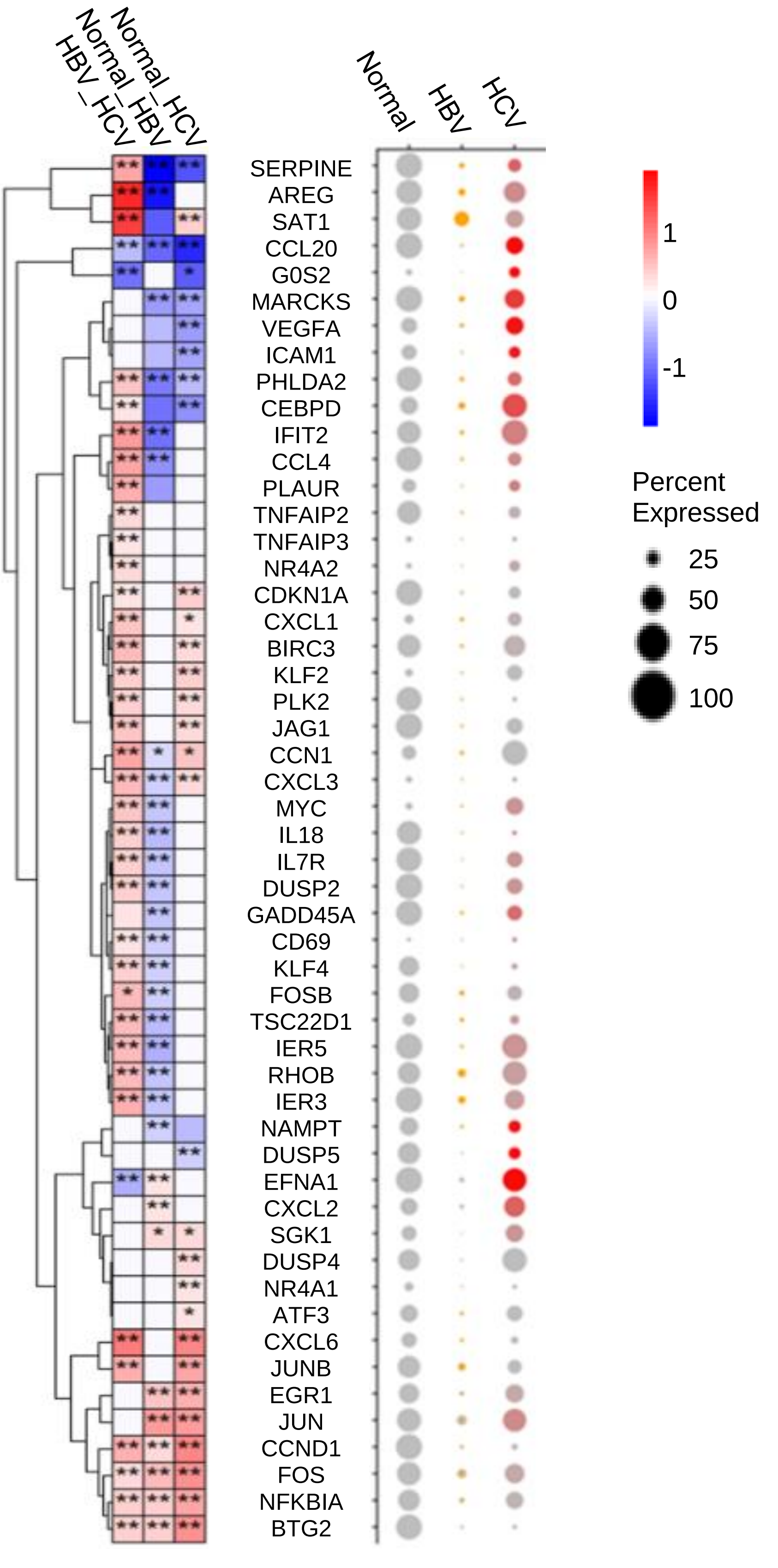

Gene expression abundance of genes involved in TNF-α signalling pathway. The left panel represents a heatmap of differential expression abundance in the TNF-α signalling pathway. Individual data are presented as fold changes relative to normal. The right panel shows the expression of genes involved in TNF-α signalling.

Supplementary Figure 6

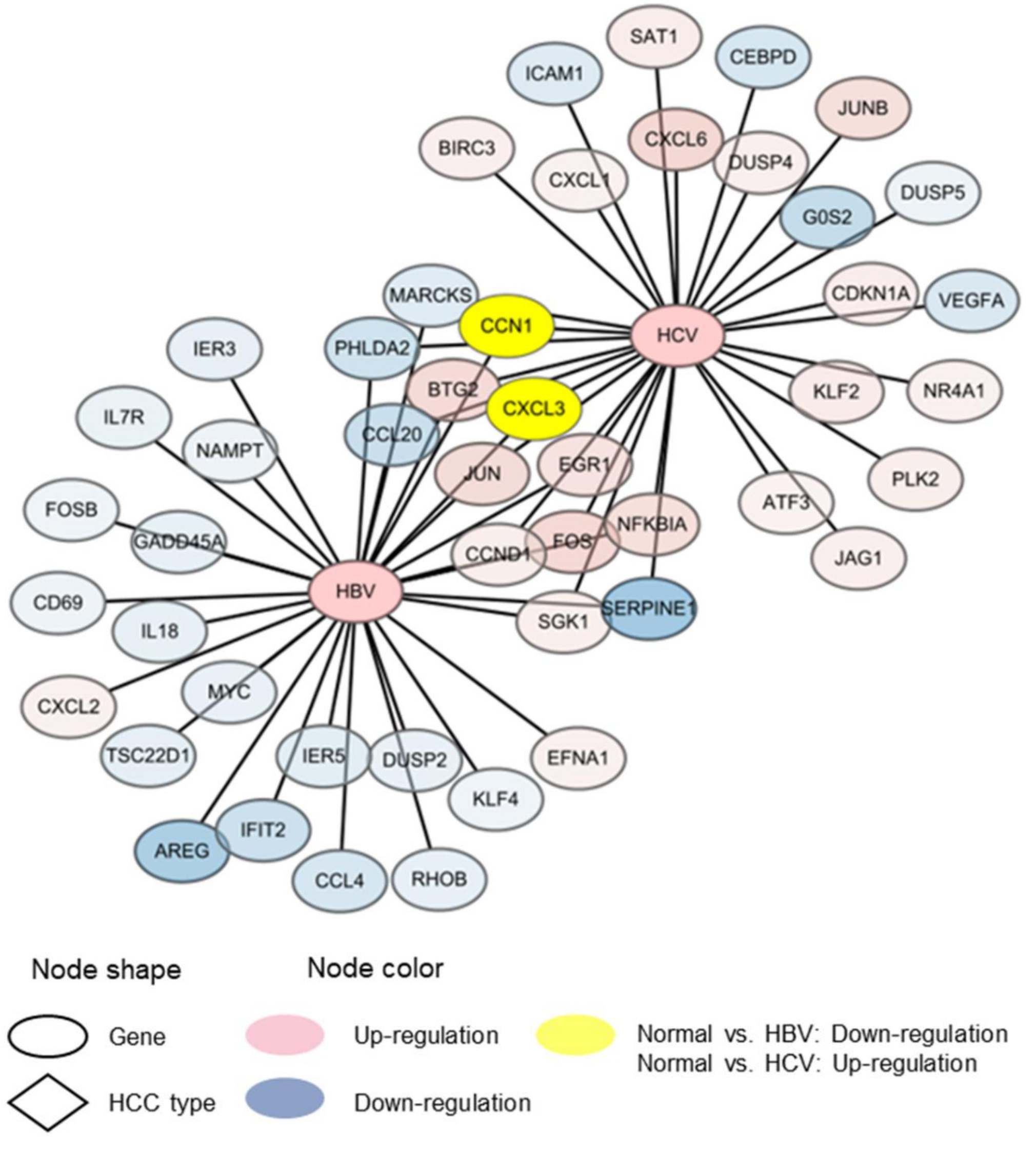

Integrated network of target genes for the TFN-α signalling pathway.

Supplementary Figure 7

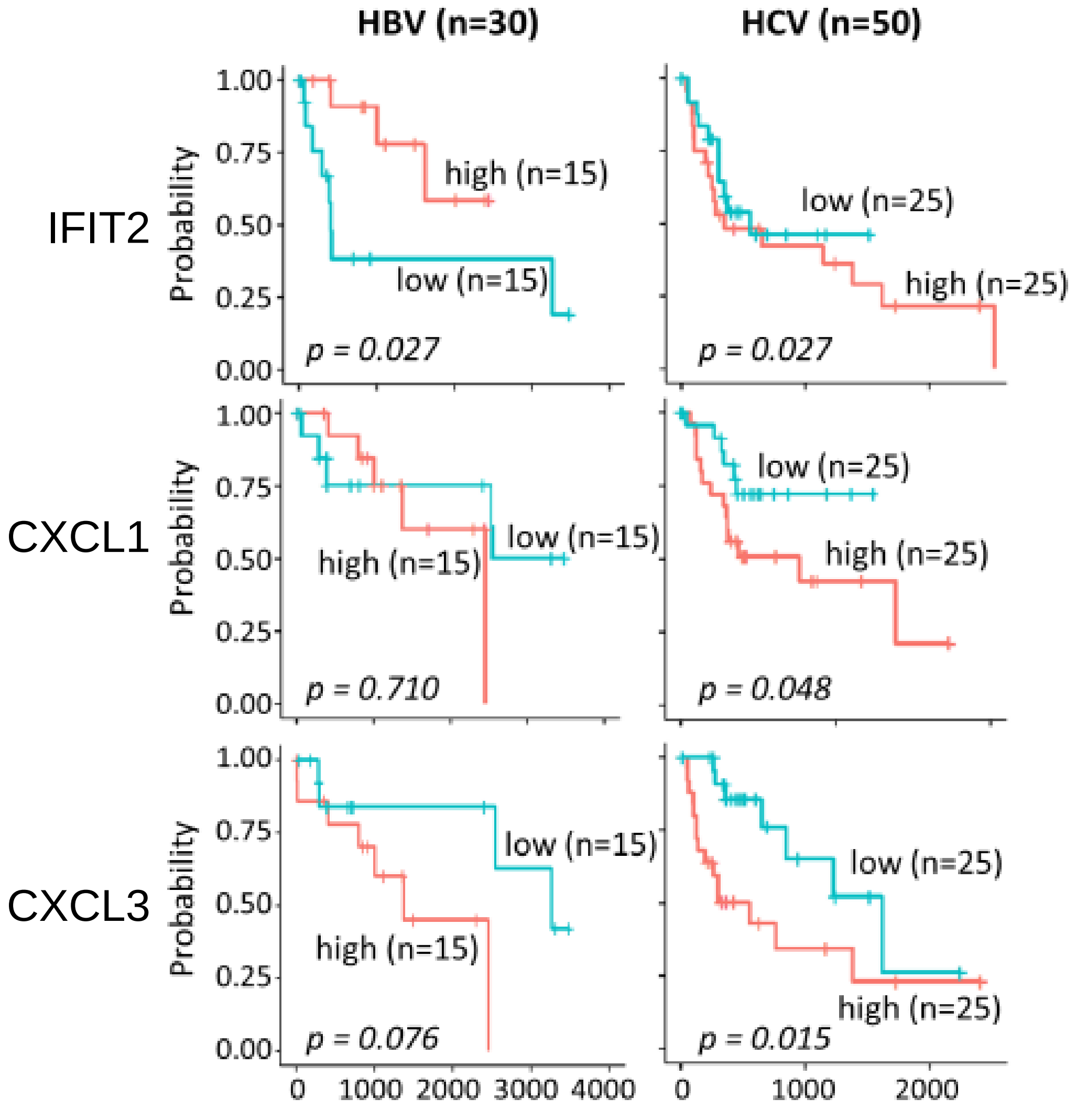

Kaplan–Meier survival curves of HBV+ and HCV+ HCC patients based on the expression of IFIT2, CXCL1, and CXCL3. The blue and red lines indicate patients with low and high gene expression, respectively.

Supplementary Figure 8

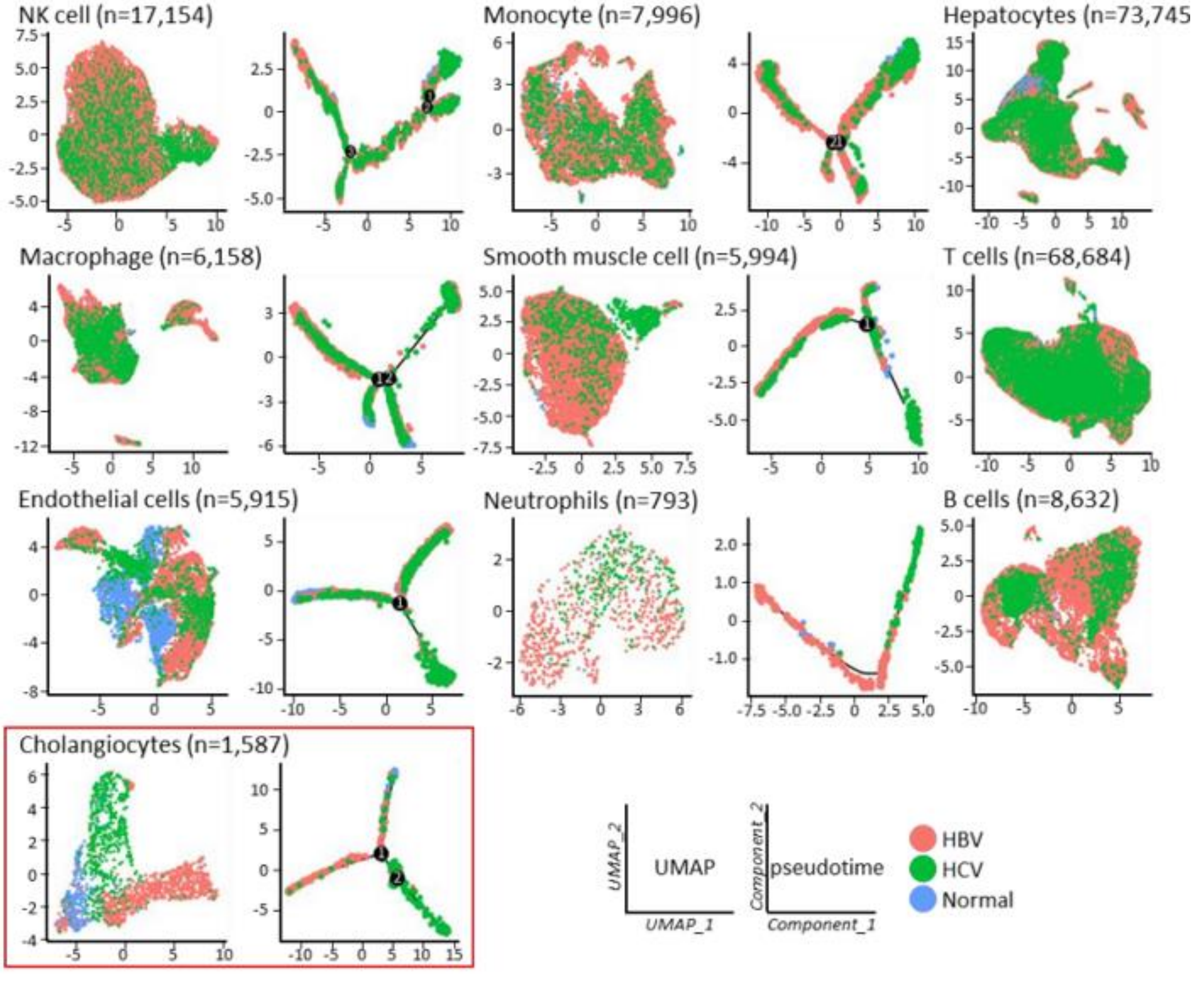

Results of UMAP clustering (left) and pseudotime analysis (right) performed on each cell type. However, pseudotime analysis of hepatocytes, T cells, and B cells could not be calculated. The red square represents the cell type we focused on.

Supplementary Figure 9

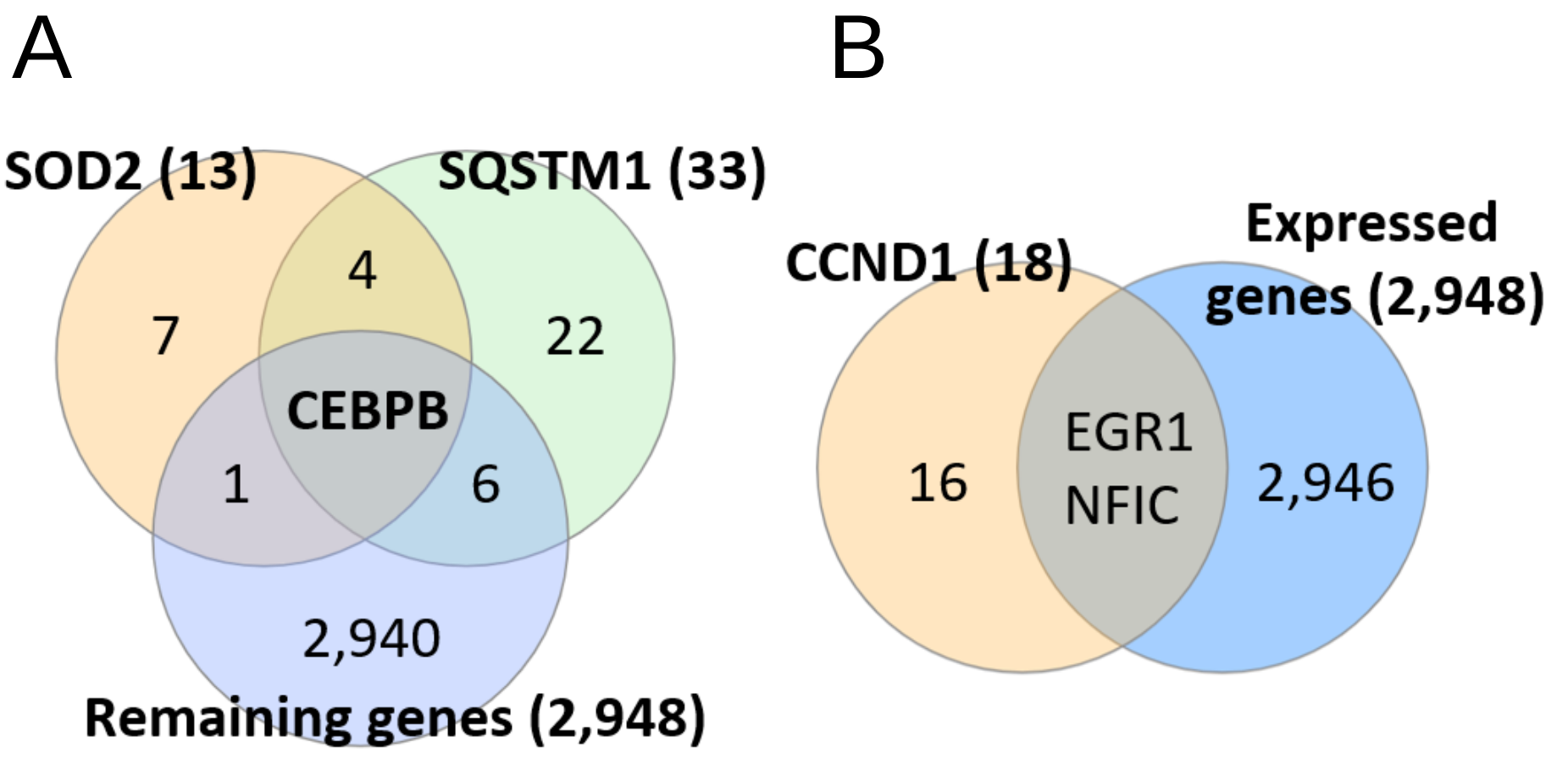

(A) Venn diagram showing the number of genes from transcription factors known to regulate the SOD2, SQSTM1. The term of 'Expressed genes' means the remaining genes except for genes that do not express more than 90% of 1,587 cells (expression count = 0). (B) Venn diagram representing the number of genes from transcription factors known to regulate CCND1. The term of 'Expressed genes' means the remaining genes except for genes that do not express more than 90% of 1,587 cells (expression count = 0). A bold gene (EGR1) means that it has the highest correlation with a specific gene among overlapped transcription factors.
