# Supplementary material for: Different regulatory mechanisms of hepatitis B- and C-positive hepatocellular carcinoma by single-cell RNA sequencing
Source: Genes Dis. 2026 Jun 9;14(2):102289. doi: 10.1016/j.gendis.2026.102289 (PMC13634556; doi:10.1016/j.gendis.2026.102289)
Supplement: Supplementary file 2 [file mmc1.docx]

**Different regulatory mechanisms of hepatitis B- and C-positive hepatocellular carcinoma by single-cell RNA sequencing**

**Materials and Methods**

*Subjects*

Tissues were also obtained from eight patients who underwent liver resection for HCC at Seoul St. Mary's Hospital from August 2018 to May 2020 and used for single-cell transcriptome analysis. Detailed sample information is described in Table S1. Additionally, we downloaded 43 public scRNA-seq samples from Gene Expression Omnibus (GEO; <https://www.ncbi.nlm.nih.gov/geo/>) and other repositories (<https://ngdc.cncb.ac.cn/gsa/>): normal (GSE115469^1^, GSE158723^2^); HBV+ HCC (CRA002308^3^, GSE151530^4^, and SRP318499^5^); HCV+ HCC (CRA002308^3^, SRP318499^5^).

*Ethical approval and consent to participate*

This study was approved by the Institutional Review Board of the College of Medicine, Catholic University of Korea (approval number KC17TESOI0008). All patients provided written informed consent prior to sample collection.

*scRNA-seq library construction and sequencing*

Single-cell suspensions were loaded onto the Chromium Single Cell Controller using the Chromium Single Cell 3′ Library & Gel Bead Kit v2 or v3.1 (both from 10X Genomics, Pleasanton, CA), following the manufacturer’s instructions. Briefly, single-cell suspension was loaded into a Chromium controller. The cells were partitioned into gel beads in emulsion (GEMs) in the Chromium controller, where cell lysis and barcoded reverse transcription (RT) of RNA occurred. The resulting post-GEM product complementary DNA (cDNA) was purified according to the manufacturer’s instructions using Dynabeads MyOne SILANE (Thermo Fischer Scientific). Library preparation, including cDNA amplification, enzymatic fragmentation, processing, and cleaning, was performed according to manufacturer’s instructions. Sequencing libraries were sequenced on Illumina HiSeq2500 platform.

*Preprocessing of scRNA-seq data*

The overall data generation process followed a pipeline provided by the 10X genomics Cell Ranger software (https://support.10xgenomics.com/single-cell-gene-expression/software/pipelines/latest/what-is-cell-ranger, v3.1.0)^6^. Cell Ranger demultiplex raw base call (BCL) files were generated using Illumina sequencers in FASTQ files with the mkfastq module. The FASTQ files were aligned to the human genome reference sequence (hg38) using the Cell Ranger count module; we generated a gene expression matrix, web summary, and aligned bam files using this module. The quality of scRNA-seq data was checked using fraction reads in cells and mapping scores in the web summary; the sequencing depth was set to > 20,000 reads per cell for each sample.

*scRNA-seq data merging and data analysis*

A total of 51 samples were integrated using the “IntegrateData” function in Seurat ^7^. To identify the shared cell types and correct batch effects between the data produced in this study and public single-cell data, we normalized single-cell level expression data using the “FindIntegrationAnchors” method in Seurat. After merging the public and generated data, all merged single-cell datasets underwent low-quality cell filtering; the Seurat R package was then used for normalizing, scaling, and clustering datasets. The global-scaling normalization method “LogNormalize” was used for each cell's feature expression measurements. Then, the “FindVariableFeatures” function identified highly variable genes and thus, significant features that highlight biological signaling data. Next, the “ScaleData” function was used to scale the expression data. The linear dimensional reduction was performed on the scaled data of highly variable genes using the “RunPCA” function. The “FindClusters” function was used to cluster the cells with the top 20 principal components at a resolution parameter of 0.5. The graph-based cell clusters were visualized using the uniform manifold approximation and projection method. The cell type of each cluster was manually annotated using marker genes selected from previous HCC papers ^5;8^.

*Data processing of differentially expressed genes*

The marker genes for each cell cluster versus all cells were computed using the FindAllMarkers function in Seurat version 4.1.1. Differentially expressed genes (DEGs) between HBV+ or HCV+ cells versus normal cells were computed using the “FindMarkers” function with the default parameters.

*Pathway enrichment analysis*

Gene set enrichment analysis (GSEA) was performed on pre-ranked values according to DEG analysis using the fgsea (v1.12.0) package in Bioconductor (<http://bioconductor.org/packages/fgsea/>) and GitHub (<https://github.com/ctlab/fgsea/>)^9^. Gene sets with hallmarks used as input were obtained from the Molecular Signature Database^10^. Significantly enriched pathways were selected based on their normalized enrichment score and at least one condition with a *p* < 0.05.

*Construction of integrated gene network in TNF-α signaling*

Of the genes involved in TNF**-**α signaling, subsets consistently upregulated or downregulated by HBV+ and HCV+ cells versus normal cells were selected. HBV+ and HCV+ effects on these genes were analyzed to construct an integrated network of key genes and visualized using Cytoscape^11^.

*Survival analysis and expression level of core genes*

Gene expression data and corresponding clinical data of patients with hepatocellular carcinoma (LIHC) were downloaded from The Cancer Genome Atlas (TCGA)^12^. To further investigate whether core genes in TNF**-**α signaling were related to HBV+ and HCV+ HCC, survival analysis was performed using the R “survival” package. A Kaplan–Meier plot was generated to assess correlations between the expression of core genes and overall survival in patients with HCC. Prognostic genes were selected using the log-rank test (*p* < 0.05).

*Trajectory analysis*

We used Monocle2^13^ to infer single-cell trajectories and place each cell at its appropriate position in the trajectory. CellDataSet (CDS) object is required for trajectory analysis. We first preprocessed Seurat objects to remove filtered cells, estimate size factor and dispersion, filter low-quality cells, and perform differential analysis. Next, setOrderingFilter, reduceDimension with max_components = 2, method="DDRTree", and orderCells were used; results were plotted using the plot_cell_trajectory function. We used the BEAM function in Monocle2 to discover branch-dependent regulated genes, along with pseudotime, at the default setting and a cut-off FDR value < 0.05.

*Transcription factor (TF) analysis*

To identify TFs regulating the selected genes, including *SOD2, SQSTM1*, and *CCND1*, we retrieved a list of known TFs for the corresponding genes from the JASPAR database^14^. Among the TFs that simultaneously regulate *SOD2* and *SQSTM1*, CEBPB was selected, for which the scRNA-seq expression data were not filtered. We calculated the Pearson correlation coefficient of each TF and the corresponding gene to confirm their significance.

*Validation of virus-dependent gene regulation*

To validate the expression levels of virus-dependent regulated genes, we retrieved the expression profile data from TCGA and GEO. A total of 322 samples containing three public datasets were used for validation from TCGA-LIHC (<https://gdac.broadinstitute.org/>) and GEO (<https://www.ncbi.nlm.nih.gov/geo/>; HBV+ HCC and HBV+ normal (GSE121248)^15^, HCV+ HCC and HCV+ normal (GSE17856)^16^). We used the Student’s *t*-test to determine significance and ggplot2 (3.4.2) in R for plotting.

**References**

1.	MacParland SA, Liu JC, Ma XZ, et al. Single cell RNA sequencing of human liver reveals distinct intrahepatic macrophage populations. *Nat Commun.* 2018;9(1):4383.

2.	Payen VL, Lavergne A, Alevra Sarika N, et al. Single-cell RNA sequencing of human liver reveals hepatic stellate cell heterogeneity. *JHEP Rep.* 2021;3(3):100278.

3.	Song G, Shi Y, Zhang M, et al. Global immune characterization of HBV/HCV-related hepatocellular carcinoma identifies macrophage and T-cell subsets associated with disease progression. *Cell Discov.* 2020;6(1):90.

4.	Ma L, Hernandez MO, Zhao Y, et al. Tumor Cell Biodiversity Drives Microenvironmental Reprogramming in Liver Cancer. *Cancer Cell.* 2019;36(4):418-430 e416.

5.	Ho DW, Tsui YM, Chan LK, et al. Single-cell RNA sequencing shows the immunosuppressive landscape and tumor heterogeneity of HBV-associated hepatocellular carcinoma. *Nat Commun.* 2021;12(1):3684.

6.	Zheng GX, Terry JM, Belgrader P, et al. Massively parallel digital transcriptional profiling of single cells. *Nat Commun.* 2017;8:14049.

7.	Hao Y, Stuart T, Kowalski MH, et al. Dictionary learning for integrative, multimodal and scalable single-cell analysis. *Nat Biotechnol.* 2024;42(2):293-304.

8.	Li XY, Shen Y, Zhang L, Guo X, Wu J. Understanding initiation and progression of hepatocellular carcinoma through single cell sequencing. *Biochim Biophys Acta Rev Cancer.* 2022;1877(3):188720.

9.	Korotkevich G, Sukhov V, Budin N, Shpak B, Artyomov MN, Sergushichev A. Fast gene set enrichment analysis. *bioRxiv.* 2021:060012.

10.	Liberzon A, Subramanian A, Pinchback R, Thorvaldsdottir H, Tamayo P, Mesirov JP. Molecular signatures database (MSigDB) 3.0. *Bioinformatics.* 2011;27(12):1739-1740.

11.	Shannon P, Markiel A, Ozier O, et al. Cytoscape: a software environment for integrated models of biomolecular interaction networks. *Genome Res.* 2003;13(11):2498-2504.

12.	Cancer Genome Atlas Research Network. Electronic address wbe, Cancer Genome Atlas Research N. Comprehensive and Integrative Genomic Characterization of Hepatocellular Carcinoma. *Cell.* 2017;169(7):1327-1341 e1323.

13.	Qiu X, Hill A, Packer J, Lin D, Ma YA, Trapnell C. Single-cell mRNA quantification and differential analysis with Census. *Nat Methods.* 2017;14(3):309-315.

14.	Rauluseviciute I, Riudavets-Puig R, Blanc-Mathieu R, et al. JASPAR 2024: 20th anniversary of the open-access database of transcription factor binding profiles. *Nucleic Acids Res.* 2024;52(D1):D174-D182.

15.	Wang SM, Ooi LL, Hui KM. Identification and validation of a novel gene signature associated with the recurrence of human hepatocellular carcinoma. *Clin Cancer Res.* 2007;13(21):6275-6283.

16.	Tsuchiya M, Parker JS, Kono H, Matsuda M, Fujii H, Rusyn I. Gene expression in nontumoral liver tissue and recurrence-free survival in hepatitis C virus-positive hepatocellular carcinoma. *Mol Cancer.* 2010;9:74.
